# By the numbers: a quantitative content analysis of children's picturebooks

**DOI:** 10.3389/fpsyg.2013.00850

**Published:** 2013-11-12

**Authors:** Laura Wagner

**Affiliations:** Department of Psychology, Ohio State UniversityColumbus, OH, USA

**Keywords:** picturebooks, story grammar, literacy, narrative, childrens books

Children's picturebooks are a distinctive art form and draw on a variety of story-telling and pictorial traditions; they are vibrant, everyday elements in the lives of many children. The dominant approach for researching picturebook content—stemming largely from the traditions in education and literary studies that have been most concerned with it—has been a qualitative one that focuses on detailed explorations of targeted books and a small number of children. This qualitative work has yielded (and continues to find) important insights. The current work, however, adopts a different approach and aims to a provide quantitative analysis of picturebooks.

## A quantitative database of picturebook content

Before arguing for the potential benefits of a quantitative approach, it is worth considering what such an approach looks like. Recently, my lab has been engaged in creating a quantitative database of picturebook content. The database is quantitative in two critical senses. First, it catalogs a reasonably large sample of books (we are starting with 100) that covers a somewhat representative sample of what children actually read. The sample includes books written since the year 2000 (*N* = 56) as well as older classics; Caldecott award winners (*N* = 14) as well as books connected to movies or TV shows (*N* = 15) and books suggested by parents in a survey (*N* = 47); narrative stories (*N* = 89) as well as thematic and ABC books; books explicitly marketed to infants 2-years of age and younger (*N* = 27) as well as books for toddlers (*N* = 55) and books aimed at older pre-schoolers (*N* = 18). The goal is to insure that the generalizations found in the sample accurately reflect the range of books available and of interest to children.

The second sense in which the database is quantitative is that the books are not only coded for categorical properties (e.g., Is the protagonist a child or an adult? What is the narrator's point of view and access to knowledge?) but in addition, every picture within each book is coded for its own properties (e.g., What character is in focus? How is the picture situated on the page?) as well as how it differs from the previous picture (e.g., Has the depicted location changed? Is the picture in the same artistic style?), and a complete hierarchical story-grammar is constructed for each book. The goal of this detailed coding is to allow for graded classifications of entire books as well as detailed examinations of within-book structure; each book can receive a quantitative profile that numerically summarizes how it embodies a range of elements. The full set of codes is compiled so that the information can be sorted and analyzed along any dimension that suggests itself as a fruitful research question. Moreover, the database is expandable and can incorporate new coding categories as needed.

## Using quantitative data for literary analyses

Conceptually, the idea of enumerating common story components as a means of understanding a genre goes back at least to Propp (1968). In his classic work, Propp identified a few dozen core narrative events that provided the structuring for (western) folk tales. Modern picturebooks are not folk tales, and may not constitute a single narrative genre. However, the database can help us identify common properties across the books, or at least, sub-sets of the books. Moreover, we can ask more quantitatively oriented questions, such as Which thematic features are most common across books? How many common components of story grammars do most books share? and How extensively do books make use of different narratological elements? These questions can help us create a nuanced taxonomy of this diverse set of books. For example, characterizing the protagonist is a core narratological function, but one that receives radically different treatment across our set of picturebooks. One measure of characterization depth is the number of pictures and pages that a book devotes to providing background information about the main character before a traditional narrative arc (organized around a conflict and resolution) begins. Our books range from providing 0 pages of character establishment (e.g., *Not a box*) to devoting more than 75% of the pages to character establishment (e.g., *No! David*). This difference has profound effects on the nature of the event structure of these books; for example, one consequence of devoting substantial space to character development, and consequently, less space to a conflict-resolution story arc, is that those arcs are not well elaborated, containing few episodes and very little sub-structure relative to books which devote less space to characterization. It appears there may be sub-genres of picturebooks that could potentially serve different functions for children.

More generally, the database can be used to evaluate theoretical claims about picturebook structure. For example, many researchers have argued strongly for the role of inter-dependence between pictures and text in picturebooks (e.g., Sipe, 1998; Nikolajeva and Scott, 2001). The idea is that the pictures are not merely illustrations of the textual content but that the two work together synergistically to contribute to meaning-making in the stories. One way to quantify this idea was proposed by Martinez and Harmon (2012). They looked at different narrative functions such as setting, plot, and mood and asked whether the information was being conveyed through the pictures, the text, both independently, or both inter-dependently. Their work found that genuine instances of inter-dependence—where the pictures and text each contributed critical and non-identical information—were not dominant within the books, never reaching higher than 20% of the instances of a narrative function. Our coding uses similar categories to Martinez and Harmon, and preliminary analyses suggest that we are replicating their results with our larger sample.

Moreover, our coding schemes have also drawn on theoretical insights from analyses of comic books (McCloud, 1993; Cohn, 2010). For example, McCloud (1993) analyzes the transitions between panels in comics in terms of how space and time relations change and convey a sense of progress. He argues that most comics (particularly western ones) rely primarily on action-to-action transitions, although experimental and Japanese comics use a somewhat more diverse set of transition types. Our preliminary analyses of picturebooks suggest that despite their traditional and western origins, they may have more in common with experimental and Japanese comics. Like those latter kinds of comics, the picturebooks appear to contain a significant minority of moment-to-moment and scene-to-scene transitions across the pictures in addition to the more common action-to-action transitions. Further investigations will examine the extent to which different transition types are linked to different sub-types of books and different narrative functions. Moreover, anticipating the next section slightly, these transition patterns suggest experimental questions about how children's interpretations of different pictorial representations of time might be related to their developing understanding of temporal concepts in general, including their linguistic expression.

From a literary standpoint, therefore, the quantitative approach complements qualitative insights and allows us to capture how books are structured and related to each other in a systematic, detailed fashion.

## Picturebooks as input for children's narrative knowledge

Comprehension of narrative structure and the ability to re-create that structure when telling stories are important, advanced linguistic skills that continue to develop throughout the school-age years. The dominant approach to characterizing narrative competence comes from the story grammar tradition (but see (Nicolopoulou, 2008) for an alternative approach). Story grammars are hierarchal schematic representations of the story that capture basic narratological functions and are generally centered around the goal structure of the characters (e.g., Mandler and Johnson, 1977; Nezworski et al., 1982). Experimental work with adults and children has supported the idea that the grammars are psychologically valid: participants remember properly structured stories better (Mandler and Johnson, 1977) and can make explicit judgments about story organization that reflect story grammar elements (Gee and Grosjean, 1984; Mandler, 1987).

Studies with elementary school children have found that it is possible to improve children's understanding of story grammars by providing training on it. For example, Stevens et al. (2010) had kindergarten and first grade teachers use an interactive protocol that explicitly talked about story grammar elements during regular story-time periods (e.g., Who is the protagonist? What is the conflict?). Compared to a control group, the children who received this protocol could remember more about the stories they heard and their improved story comprehension extended to improved performance on a standardized reading test.

But even children who don't receive explicit training in story grammars still often come to understand and tell appropriate stories; most children implicitly learn about the underlying structures from the stories they hear. Indeed, children whose parents read to them more frequently show advantages in early language and literacy skills (Fletcher and Reese, 2005). Since what parents read to young children are often picturebooks, these books are one of the core input sources for children to learn about story structure from. Understanding which aspects of these books facilitate (or hinder) children's narrative understanding is useful for educators, and potentially, to authors themselves.

With the quantitative analysis of the books themselves, my lab is asking what information about story grammar is available for children to use: what components of story grammars are typically present and how much of each book is devoted to conveying the key story elements? Once these analyses are in place, we are positioned to ask how children themselves respond to different patterns in their input. For example, preliminary analyses of the story grammars of the sample have found that some books contain a very traditional narrative arc centered around a conflict and its resolution, and explicitly instantiate almost all the major elements of story grammars (e.g., *Caps for Sale)* while in other books, one or more major story grammar elements must be inferred (e.g., the primary central event of *Madeline*, the operation to remove her appendix, must be inferred from context) and other books simply lack a causal story arc altogether (e.g., *Maisy Goes to Preschool*). One might hypothesize that children at various levels of narrative competence would find these different types of books differentially engaging; further, one might predict that the books could be sequenced to provide optimal scaffolding for children's narrative development.

Another research question concerns how children make use of the picture and text information within a picturebook. Eye-tracking work by Justice et al. (2005) found that pre-school aged children devote nearly all of their attention to examining the pictures in picturebooks as opposed to looking at the text. This finding suggests the hypothesis that children will comprehend books better when the main story elements are conveyed through the pictures; or perhaps even within an individual book, children will comprehend those specific story elements that are conveyed via pictures better than those conveyed via text. Moreover, the field of cognitive development offers an alternative suggestion for how children might make best use of picture-text relations. Studies investigating how children interpret gestures that accompany speech (Goldin-Meadow et al., 1993) have found that in many cases, children learn better when the two sources convey different pieces of information; that is, when there is a mis-match between gesture and speech. Pictures and text in picturebooks may operate in an analogous fashion and it is possible that mis-matches between the two—especially for a narrative element children are in the process of mastering—could constitute a critical scaffolding cue for improving narrative ability. Using the database, we can readily identify books that convey different story elements in different ways and select the critical cases to examine children's comprehension of story structure.

## Conclusions

Picturebooks are a rich object of study in their own right and a fertile domain in which to identify specific hypotheses about what might drive children's narrative development. A detailed, quantitative analysis of these books not only allows for new insights into the structure of the books themselves, but it supports a range of specific hypotheses about how these books can influence children's ability to understand and tell good stories.

## Picturebooks mentioned

Bemelmans, L. (1960). *Madeline.*

Cousins, L. (2009). *Maisy Goes to Preschool.*

Portis, A. (2007). *Not a Box.*

Shannon, D. (1998). *No, David!*

Slobodkina, E. (1938). *Caps for Sale.*
